# History of the discovery of a master locus producing piRNAs: the *flamenco*/*COM* locus in *Drosophila melanogaster*

**DOI:** 10.3389/fgene.2014.00257

**Published:** 2014-08-04

**Authors:** Goriaux Coline, Emmanuelle Théron, Emilie Brasset, Chantal Vaury

**Affiliations:** ^1^Laboratoire GReD, Faculté de Médecine, Clermont Université – Université d’Auvergne, Clermont-FerrandFrance; ^2^INSERM, U 1103, Clermont-FerrandFrance; ^3^CNRS, UMR 6293, Clermont-FerrandFrance

**Keywords:** *flamenco*, *COM*, piRNA cluster, transposable elements, β-heterochromatin, *Drosophila melanogaster*

## Abstract

The discovery of transposable elements (TEs) in the 1950s by B. McClintock implied the existence of cellular regulatory systems controlling TE activity. The discovery of *flamenco* (*flam)* an heterochromatic locus from *Drosophila melanogaster* and its ability to survey several TEs such as *gypsy*, *ZAM,* and *Idefix* contributed to peer deeply into the mechanisms of the genetic and epigenetic regulation of TEs. *flam* was the first cluster producing small RNAs to be discovered long before RNAi pathways were identified in 1998. As a result of the detailed genetic analyses performed by certain laboratories and of the sophisticated genetic tools they developed, this locus has played a major role in our understanding of piRNA mediated TE repression in animals. Here we review the first discovery of this locus and retrace decades of studies that led to our current understanding of the relationship between genomes and their TE targets.

In the 1950s, Barbara McClintock first discovered transposable elements (TEs) by analyzing genetic stocks of corn that were phenotypically unstable. Her discovery implied that a genetic control exerted by genomes was generally used to regulate TE mobilization. Any loss or decrease of this control would consequently result in severe genetic instabilities due to mobilization of TEs. Just such a genetic instability affecting the genome of *Drosophila melanogaster* under the control of a locus called *flamenco* (*flam*) was first reported in 1983. Focused on *flam*, this review retraces the numerous studies that have been performed from its discovery to the understanding of its ability to survey TEs.

## A SINGLE GENOMIC MUTATION IS RESPONSIBLE FOR *Gypsy* ACTIVITY, A RETROELEMENT FROM *Drosophila melanogaster*

In the 1980s, Busson et al. (1983) were studying the dominant *ovoD* mutation in *D. melanogaster* . The *Drosophila ovo* gene, which encodes a putative transcription factor (Ovo) with TFIIIA-like zinc fingers, is required for female germline survival and proper oogenesis. The gain of function *ovoD* allele results from an extension of the N-terminal region which gives rise to a neomorphic protein that causes female sterility (Mevel-Ninio et al., 1996). Busson et al. (1983) performed crosses between *OvoD* males and females from a stock of flies from the lab of Madeleine Gans (MG) carrying a *y v f mal* X-chromosome . In the progeny, reversions of the *ovoD* mutation generating recessive *ovo* alleles were frequently observed which allowed fertile daughters to be recovered. Surprisingly, these reversions were also associated with the appearance of mutations in other loci, which could potentially be explained if such crosses were accompanied by the *de novo* mobilization of TEs. Mével-Ninio et al. (1989) found that, indeed, a high frequency of *gypsy* insertions was observed in the progeny of this cross and that a hot spot for *gypsy* exists into the *ovo* locus . Insertions of *gypsy* into the *ovo* locus interfere with the coding sequence of the neomorphic allele resulting in a null allele of the gene. Novel gypsy insertions can thus be assayed by the presence of fertile daughters. The gypsy mobilization could then explain both the genetic instability observed in these crosses and the *ovoD* reversion.

Also, Kim et al. (1989) reported a mutator strain (MS) of *D. melanogaster* characterized by an elevated frequency of spontaneous mutations in the germ line up to 10^-3^ - 10^-4^. Mutations were recovered in both sexes and displayed the characteristics of being unstable with frequent reversion to wild type or to new mutant states. When analyzing the localization of a battery of TE families, they found that the genomic distribution of *P*, *mdg1*, *412* (*mdg2*), *mdg3*, and *copia* did not vary among the individuals of this strain. However, this was not the case for *gypsy* (*mdg4*) whose frequency of transposition was high and copy number greatly increased to 30–40 copies.

These initial studies identified different mutator lines in which the frequency of *gypsy* insertions is high while several other TE families remain stable (Mével-Ninio et al., 1989; Kim et al., 1990; Lyubomirskaya et al., 1990). Further work ultimately showed that these *gypsy* instabilities within MS strains resulted from the combination of two factors: the presence of transpositionally active *gypsy* copies, and mutation(s) of loci regulating their transposition (Kim et al., 1994). These early studies provided an incredible powerful tool to evaluate *gypsy* activity by assessing the occurrence of fertile females resulting from *ovoD* reversion to a null allele. With the *ovoD* fertility test, one could isolate rare events without having to deal with enormous amount of progeny to score. Interestingly, these tools were created even before the understanding of the mechanism of repression.

## A β-HETEROCHROMATIC LOCUS CONTROLS SEVERAL RETROELEMENTS: *gypsy*, *ZAM*, AND *Idefix*:

A mutation responsible for *gypsy* mobilization was identified within the y ***v*** *f mal* chromosome of MG stocks (Prud’Homme et al., 1995). Genetic mapping localized this mutation at the basis of the X-chromosome at position 65.9 (20A1-3) close to β-heterochromatin where numerous TEs were known to accumulate (Vaury et al., 1989). The locus was called *flamenco* (*flam*) because it had the ability to make *gypsy* “dance.” Non-permissive or permissive alleles of *flam* were defined according to their ability to restrict or allow *gypsy* mobilization, respectively. A fine-scale analysis of *flam* genetic characteristics uncovered that: (i) Its control on *gypsy* activity occurs under a strict maternal effect since transposition is only allowed in the progeny (male and females) of homozygous permissive females even if fathers are non-permissive. (ii) The mutant allele present in the MS strains is essentially recessive. (iii) Transposition is largely a premeiotic event. (iv) Although *ovoD* reversion is primarily controlled by *flam*, it is influenced by other factors such as age and temperature, reversion being higher in young flies grown at 25°C. (v) The effects of *flam* on *gypsy* expression are restricted to the somatic follicle cells that surround the maternal germline (Pelisson et al., 1994). Thus, *flam* function could be viewed as the maternal transmission of some factors preventing *gypsy* transposition.

**FIGURE 1:**
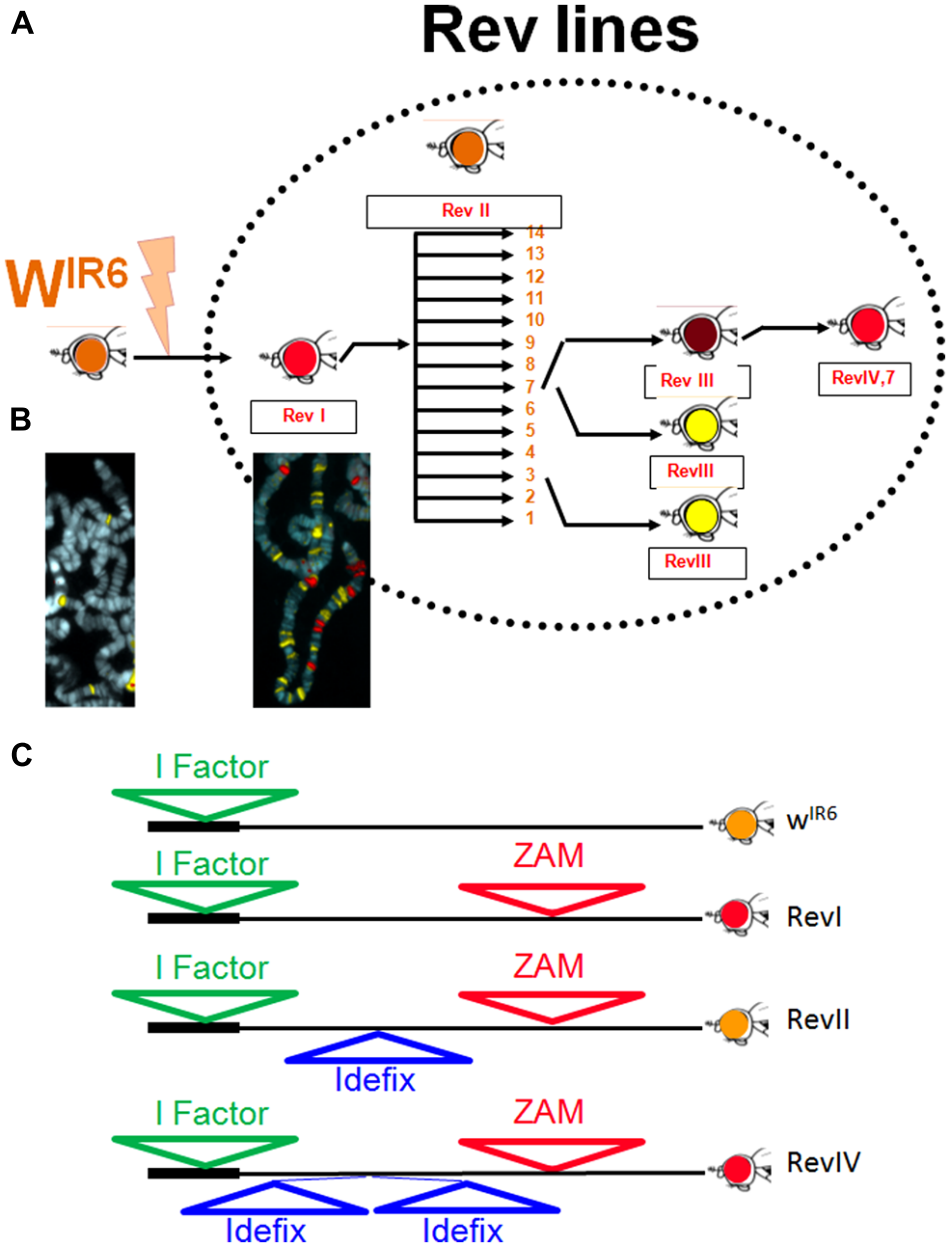
**The Rev line: **(A)** History of the unstable line, Rev, recovered after a PM mutagenesis performed on the *w*^**IR6**^ line.** In Rev, recurrent mutations affecting the eye color are recovered giving rise to derived lines successively called RevI, RevII, RevIII, and RevIV. **(B)** FISH mapping of *ZAM* (red) and *Idefix* (yellow) in w^IR6^ (left) and Rev (right). **(C)** Molecular structure of different alleles of the *white* gene recovered in the Rev lines.

In 1997, an unstable line called Rev was recovered after a PM mutagenesis performed on the line bearing the *w^IR6^* allele (Leblanc et al., 1997; Figure 1A). The *w^IR6^* allele is due to the insertion of the non-LTR retrotransposon *I-factor* into the first intron of the *white* gene. It gives an orange eye phenotype to flies (Lajoinie et al., 1995). From the PM mutagenesis (Robertson et al., 1988), a fly with a wild-type red-eye phenotype was recovered and established as a line subsequently called Rev because of the eye phenotype reversion from orange to red. It was found that the *white* locus had suffered a 8.4 kb insertion 3 kb upstream from the *white* start site of transcription (TSS; Figure 1C). This insertion corresponded to a novel TE from the *gypsy*-family that was previously uncharacterized and that has been named *ZAM* (Leblanc et al., 1997). *ZAM* did not only insert upstream of *white*. *In situ* hybridization and Southern analyses performed on the Rev genome revealed the presence of some 20 copies of *ZAM*, whereas *ZAM* was not found on the chromosomal arms of the original parental line *w^IR6^* (Figure 1B; Desset et al., 1999). From Rev, a series of mutations affecting eye coloration has been recovered, most of them affecting the *white* locus (Figure 1A). This second event of mutation resulted from the insertion of a novel *gypsy-like* transposable element designated *Idefix* that inserted 1.7 kb upstream of the TSS of the *white* gene. This second mutational event was recovered as a recurrent specific mutation in 11 independent individuals (Figures 1A,C; Desset et al., 1999). Genome analysis of Rev revealed that this line also suffered a recent and massive invasion of *Idefix* (Figure 1B).

The Rev line brought to light a new genetic model in which the activity of two TEs, *ZAM,* and *Idefix*, could be tested. Thereafter, transgenic flies were established with sensor-transgenes containing the full-length long terminal repeat (LTR) of *ZAM* or *Idefix* linked to the *LacZ* reporter gene. These transgenes provided a convenient read-out for analyzing the control exerted on these elements. Crosses designed to test the influence of the genetic background on these reporter constructs indicated that *ZAM* and *Idefix* responded to two types of controls: one restricting their expression to specific somatic cells of the ovaries and the other silencing their expression in the majority of *Drosophila* lines with only one exception reported in 2003 as being the Rev line (Desset et al., 2003).

**FIGURE 2:**
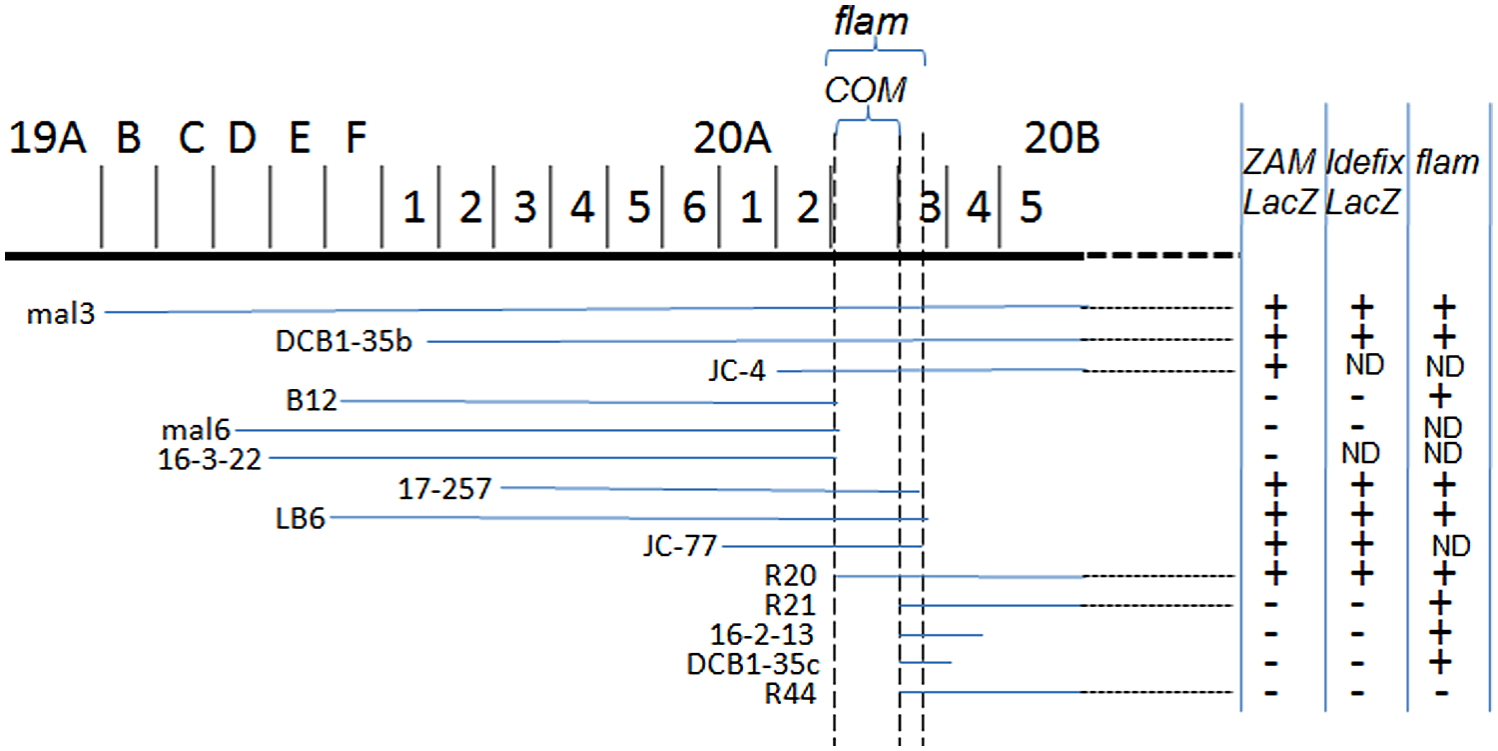
**X-chromosomal deficiencies used for cytogenetic mapping of *COM*.** The chromosomal region is presented at the top. The lines below indicate the deficiencies tested. LacZ staining observed in these lines when *ZAM-LacZ* and *Idefix-LacZ* reporters were tested are indicated on the right. Data reported for *flam* by Prud’Homme et al. (1995) are indicated in the third column. Figure modified from Desset et al. (2003).

Using these tools, a mutation responsible for the high activity of *ZAM* and *Idefix* was identified in Rev. This mutation was localized at the basis of the X-chromosome close to *flam* (Figure 2; Desset et al., 2003). Although the mutation was genetically close to *flam*, the Rev line displayed a non-permissive allele of *flam* since *gypsy* was not active in this line and, like in non-permissive lines, only few copies of *gypsy* were detected in Rev. In addition, transgenes carrying fragments of *gypsy* fused to *LacZ* used as reporters of *flam* permissivity were repressed in Rev while *ZAM-LacZ* and *Idefix-LacZ*, reporter transgenes were activated (Tcheressiz et al., 2002; Desset et al., 2003). These findings suggested that *gypsy* regulation was genetically separable from *ZAM* and *Idefix* regulation, and that a second locus existed near *flam* that controlled the activity of *ZAM* and *Idefix*.

In , while working on the silencing of testis-expressed Stellate genes by paralogous Su(Ste) tandem repeats in *Drosophila*, Aravin et al. (2001) had reported that double-stranded RNA-mediated silencing might provide the basis for negative control of gene expression. They further proposed that the related surveillance system was implicated in the control of retrotransposons in the germline (Aravin et al., 2001, 2003). Around the same time, Volpe et al. (2002) had published that double strand RNAs (dsRNAs) of centromeric heterochromatin repeats in *Schizosaccharomyces pombe* would produce small interfering RNAs (siRNAs) triggering gene silencing and repressing their own transcription (Volpe et al., 2002). They also suggested that these dsRNAs might silence other loci with homologous sequences. Therefore, we proposed a new hypothesis to account for TE regulation by the heterochromatin region at the base of the X-chromosome whereby vestiges of TEs might produce dsRNAs required for the silencing of *ZAM* and *Idefix* (Desset et al., 2003). To illustrate its potential to control over multiple TE families, we referred to this locus as a center required for TE mobilization and proposed to call it COM (Center Organisant la Mobilization; Desset et al., 2003).

Sarot et al. (2004) reported an additional finding confirming this primary model. Their study demonstrated that *gypsy* did not contain a single binding region for a putative *flam* repressor (Sarot et al., 2004). They first tested whether the *gypsy* promoter is dispensable for this regulation and swapped it for an alternative promoter from the *yp3* gene expressed in the follicle cells of the ovaries where *gypsy* itself is expressed. They found that a small 59 nucleotide fragment of non-promoter transcribed sequences was sufficient to make a non-*gypsy*-driven transcript sensitive to this regulation. They, then, tested diverse fragments between base 329 and 1072 from the *gypsy* promoter in the same way. They found that any fragment from the *gypsy* 5′-untranslated region (UTR) appeared to be able to target the repression, the only requirement being that *gypsy* sequences were present within the tested transcript. In addition, *gypsy* repression was impeded by *piwi* mutations. Short RNAs from 25 to 27 nucleotides long were also detected. These small RNAs, homologous to sequences within the *gypsy* 5′ UTR, should be able to guide RNA silencing complexes to *gypsy*-containing transcripts. In line with growing body of evidence implicating RNA silencing mechanisms in regulating TE activity, these data supported that *flam* could possibly act through a RNA-dependent mechanism.

## *flam*: FROM MOLECULAR STRUCTURE TO GENOMIC FUNCTION

Cloning of the heterochromatic locus where *flam* and *COM* had been identified proved to be very difficult. Uncertainty in the assembly of repetitive DNA in the early releases of the *D. melanogaster* genome sequence posed difficulties for heterochromatin studies. As a consequence, *flam* localized to a sequencing gap in the Release 1 genome sequence (Adams et al., 2000; Myers et al., 2000). The group of Alain Pélisson and Alain Bucheton worked very hard in tackling this locus, whose location close to heterochromatin makes its analysis extremely difficult because it is almost impossible to perform meiotic recombination. Furthermore, the repetitive nature of *flam* added to the lack of a discrete transcript produced from the locus prevented the choice of a probe that could have been used to probe cDNA libraries. A helpful tool was provided when N. Prud’Homme generated a P-element-induced mutation *P[lyB]* of *flam*. Indeed, ∼100 kB of the genomic DNA flanking the insertion could be analyzed (Robert et al., 2001). Robert et al. (2001) searched for unique sequences that might account for the activity of a gene and identified four of them with transcription units. The closest gene from the P-element insertion, *DIP1*, was assumed to be the best candidate for *flam*, notably because of its double stranded RNA-binding domains. However, all attempts to correlate its function to *gypsy* regulation proved to be unsuccessful (Robert et al., 2001). Robert et al. (2001) further detected some deficiencies permissive for *gypsy* mobilization located >130 kb away from the P-element insertion, suggesting that sequences responsible for the *flam* function lie large distances away from each other. This lab generated two new alleles of *flam* called *flam* KGP and *flam* BGP. By contrast to the *COM* mutation present in the Rev line, these new alleles brought evidence that certain *flam* mutations have the potential to relieve repression exerted not only on *gypsy* but also on *ZAM*. This study further showed that beyond its function on TE control, *flam* was required somatically for morphogenesis of the follicular epithelium, the tissue where *ZAM*, *Idefix,* and *gypsy* were repressed (Leblanc et al., 1997; Tcheressiz et al., 2002; Mevel-Ninio et al., 2007). These findings indicated that *flam* and *COM* were not always separable, and were in fact a single genomic locus (that will now be referred as *flam*) displaying flexibility in its potential to repress different TE families.

A detailed sequence of the TE content in the *flam* region became possible due to improved genome sequence data (Celniker et al., 2002) and the development of high-resolution TE annotation pipelines (Quesneville et al., 2005; Bergman et al., 2006). *flam* revealed to be one of the specific regions of the genome with an extremely high local TE density containing 104 different TE insertions from 42 different TE families spanning at >200 kb of sequence. However, because the high TE density region in the *flam* locus contained a gap in the assembly, the full structure of this locus and its TE content could not be fully determined. Nevertheless, since clear hallmarks of recurrent transposition were detected, inherent mobility of TEs was proposed to explain the high density of TEs in the *flam* region. However, a relatively high incidence of duplicated TE sequences was also identified, suggesting that segmental duplications have played a role in the genesis of the *flam* region. In line with the earlier models, the analysis of global nesting relationships among different TE families led Bergman et al. (2006) to propose that expression of chimeric sequences from regions of high TE density in the β-heterochromatin may simultaneously co-suppress transcripts from multiple euchromatic TE families .

A significant breakthrough for *flam* function was achieved in 2007 when Brennecke et al. (2007) reported for the first time the existence of discrete small RNA-generating loci that included *flam*. These data were obtained when Brennecke et al. (2007) analyzed the control of TEs and its relationship with the Argonaute proteins in *Drosophila*. Three Argonaute proteins, the PIWI proteins Piwi, Aub, and Ago3 had been shown to bind small RNAs (Liu et al., 2004). Their mutation was known to affect TE control. Sequencing small RNAs bound by each of these three PIWI proteins from *Drosophila* ovaries, Brennecke et al. (2007) found that the majority of the so-called piRNAs were derived from discrete genomic loci including *flam* that were subsequently referred to as piRNA clusters. Among piRNA clusters, *flam* displayed some unique characteristics. First, 94% of its uniquely mapping RNAs were Piwi partners. Second, *flam* produced piRNAs with a marked strand asymmetry that correlated with the strong biased orientation of TEs in the locus. Third, *flam* displayed the potential to produce a high fraction of repressive piRNAs targeting *ZAM*, *Idefix,* and *gypsy* (79, 30, and 33% respectively). The use of *flam* mutations, P(KG00476) and P(BG02658), indicated that a substantial reduction in piRNAs that uniquely map to *flam* was observed in mutant flies whereas piRNAs derived from other piRNA clusters were unaffected. This reduction of *flam* piRNAs was accompanied by a loss of *flam* transcripts and a high increase of the *gypsy* retroelement transcription.

From this piRNA sequencing, Brennecke et al. (2007) proposed that in ovaries, a pool of primary piRNAs is processed from long single-stranded transcripts encoded by piRNA clusters. These primary piRNAs target sense-transcripts encoded by TEs thereby triggering their degradation. An amplification system starting once the sense transcript has been detected by the primary piRNAs results in production of secondary piRNAs. In their turn, these secondary sense-piRNAs enhance cleavage of anti-sense precursors resulting in amplification of piRNA production. This model has been called the ping-pong model.

Although a big step in the understanding of piRNA origin had been made, the model needed to be refined to take into account that piRNAs had been extracted from a mixture of somatic and germ line cellular lineages. *ZAM*, *Idefix,* and *gypsy* had indeed been shown to be active and consequently repressed by *flam* only in the somatic follicle cells (Pelisson et al., 1994; Leblanc et al., 2000; Tcheressiz et al., 2002). In their study, Brennecke et al. (2007) noticed that the amplification cycle detected in ovaries might not operate in somatic follicle cells where Aub and Ago3 were absent. They suggested that, since the vast majority of transposon fragments within *flam* exists in a common orientation, this could lead to the production of anti-sense primary piRNAs processed from a long, unidirectional, precursor transcript. Subsequently, Malone et al. (2009) sought to determine whether the ping-pong model applied or not in both ovarian germ and somatic follicle cells. By comparing piRNAs from germline and from their somatic support cells, they found distinct piRNA pathways with differing components (Malone et al., 2009). A simplified piRNA pathway operates in the somatic lineage in which among the three Argonaute proteins, only Piwi functions. Only primary piRNAs that lack the ping-pong amplification cycle are expressed in these cells (Ishizu et al., 2012).

**FIGURE 3:**
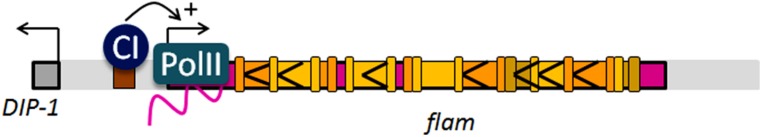
**Molecular structure of the *flam* locus.** The CI binding site, the transcription start site and the strong biased orientation of TEs indicated by arrows are schematized.

From these studies, it emerged that *flam* was not a classically defined gene producing messenger RNAs with large open reading frames able to encode proteins. By contrast, it had the potential to produce long, unidirectional, non-coding, precursor transcripts containing multiple TE families traversing the locus (Figure 3; Brennecke et al., 2007; Malone et al., 2009). Thus, although the reason why different lines might display different TE targeting remained elusive, it was then clear that the whole >180 kb of the *flam* locus could be required to generate piRNAs and to perform multiple TE surveillance.

Subsequent studies have indicated that piRNA biogenesis requires many other factors than these long TE-containing transcripts and the PIWI proteins. Thus, exhaustive screens were performed to uncover the full repertoire of genes involved in this pathway. *flam*-mediated TE control became the ideal genetic model to validate candidate genes and to elucidate their activity. Indeed, the precise heterochromatic localization of *flam* had been defined from numerous genetic approaches; several of its TE targets were well known like *gypsy*, *ZAM,* and *Idefix*; transgenic tools targeted by *flam* had been constructed; several *flam* alleles with distinct suppressions of either target control were available. To date, numerous studies can be cited in which *flam* has been used to test any gene of interest for its involvement in the somatic piRNA pathway. As few examples see: Saito et al. (2009, 2010), Haase et al. (2010), Qi et al. (2010), and Muerdter et al. (2013).

## *flam* TRANSCRIPTION GENERATES DIVERSE RNA PRECURSORS BEFORE BEING PROCESSED INTO piRNAs

Although it provided a useful tool to validate candidate genes involved in the piRNA pathway, the mechanism of *flam* transcript did not receive much attention after the sequence analysis of its structure and piRNA production has been reported. For several years, the prevailing model held that the *flam* locus is transcribed as a continuous single stranded RNA spanning >180 kb. However, this precursor had only been detected through quantitative RT/PCR using primer pairs spanning different regions of *flam* (Brennecke et al., 2007; Haase et al., 2010). In 2010, several studies identified Yb-bodies, cytoplasmic structures close to the nuclear membrane of the follicle cells, as sites of primary piRNAs biogenesis (Olivieri et al., 2010; Qi et al., 2010; Saito et al., 2010). piRNA intermediate-like molecules (piR-ILs) of length varying between 25 and 70 nucleotides were isolated from these structures (Saito et al., 2010). They proved to be intermediate molecules between a long precursor whose structure and regulation were still unknown, and mature piRNAs.

An important issue that remained to be addressed to go further in *flam* function was to elucidate its transcriptional regulation. Rangan et al. (2011) reported that repressive marks deposited by dSETDB1were required for transcription from all major piRNA clusters including somatic unidirectional clusters like *flam*. In that, dSETDB1 was required for somatic TE control by *flam*. ChIP-seq experiments further indicated that *flam* is actively transcribed by RNA polymerase II and is fairly devoid of the histone mark H3K9me3, a marker of heterochromatic regions (Sienski et al., 2012). In 2014, new insights into *flam* activity were reported by our group (Goriaux et al., 2014). We identified the promoter of *flam* as an Inr DPE promoter located at 21 502 918, 1743 bp proximal from *DIP1* (flybase version FB2011_08) and showed that its transcriptional activity requires the transcription factor, *Cubitus interruptus* (CI; Figure 3). In addition, we found that the *flam* precursor transcript undergoes differential alternative splicing to generate diverse RNA precursors. The intron sizes are extremely diverse and range from 0.7 to 158 kb but the first exon (exon1: 21,502,918 to 21,503,349) was found to be constitutively expressed since it is always present within the processed RNAs. Furthermore, when publicly available RNA-seq libraries were interrogated (Sienski et al., 2012), piRNAs corresponding to the predicted spliced exon1–exon2 junction were identified. At the same time, piRNAs encompassing exon1/intron1 junction were under-represented in the libraries compared to piRNAs matching the spliced junction. These data indicate that *flam* transcripts are spliced before being processed in piRNAs.

**FIGURE 4:**
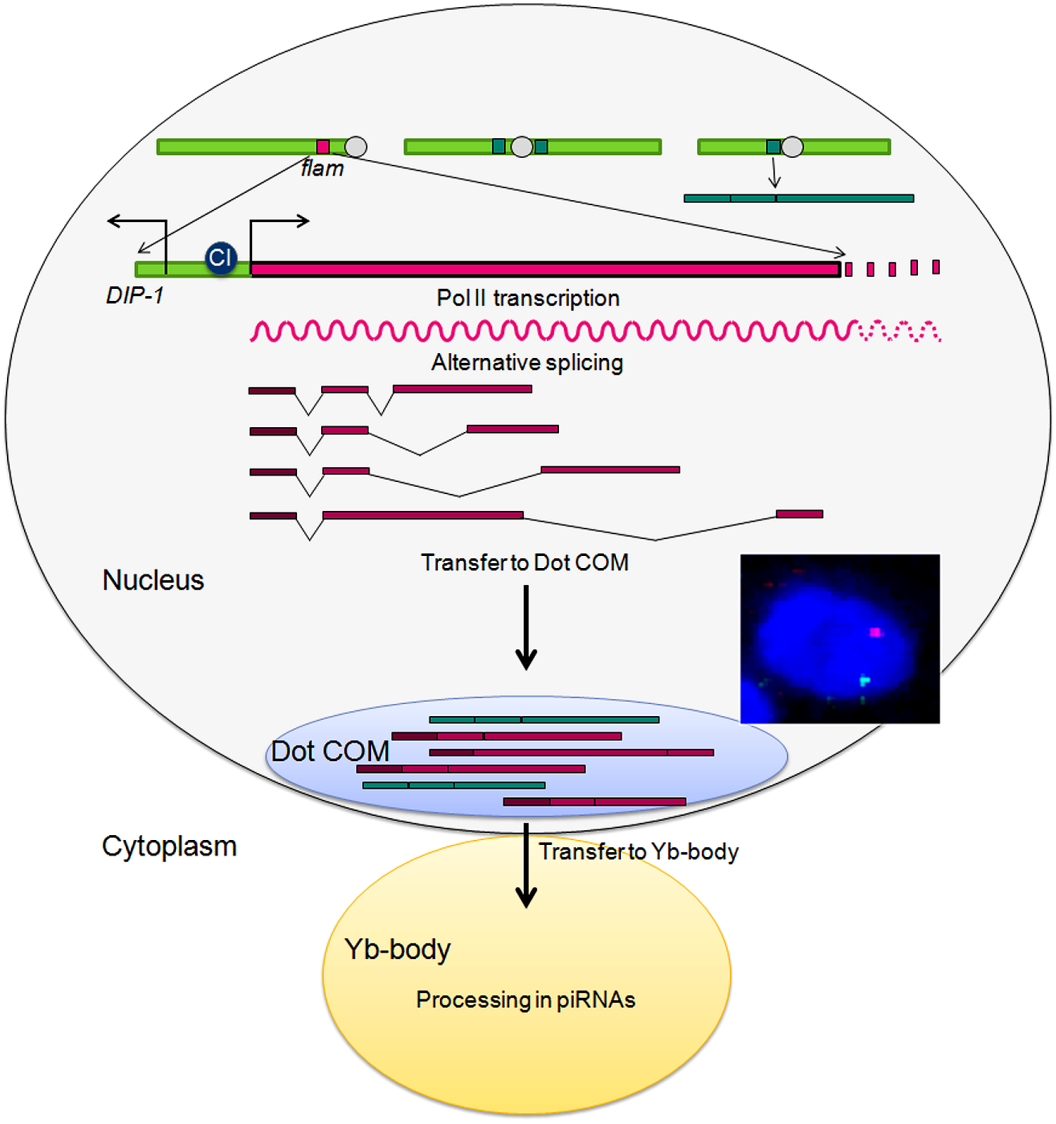
**Model of the piRNA pathway in the follicle cells of *Drosophila* ovaries.** A typical DNA/RNA immunoFISH staining with *flam* RNA in green, *flam* DNA in red, and DNA in blue is presented.

RNA FISH experiments indicated that these spliced transcripts are then transferred to the nuclear membrane. Indeed, we further identified a prominent nuclear structure called Dot COM, in which precursor transcripts encoded by *flam* accumulate (Dennis et al., 2013). Remarkably, this structure is often juxtaposed with Yb bodies and concentrates transcripts from other piRNA clusters. When Yb-bodies are disrupted using mutations of the Armi-Piwi-Yb complex composing Yb-bodies, Dot COM is normally distributed within the nucleus and its morphology unchanged. Overall these last findings suggest the following scenario: at the initial step, *flam* RNA polymerase II transcription is activated by CI in the follicle cells. Transcripts are differently spliced to form a population of RNAs along the >180 kb region but having in common the presence of the first exon. These RNAs are channeled from their site of transcription to Dot COM at the nuclear membrane in a location facing the Yb-bodies. From here, they are transferred to the cytoplasmic Yb-bodies and processed in piRNAs which in turn *trans*-silence complementary TEs located outside of *flam* (Figure 4). At this stage many questions remain to be elucidated: Where does the splicing occur? Can it be co-transcriptional or does it occur in Dot COM? How RNAs are transported from their genomic clusters to Dot COM and then to their piRNA processing center? which factors are required for these processes?

## A HIGH DEGREE OF STRUCTURAL VARIATIONS AFFECTING THE *flam* LOCUS IMPACTS THE GENOMIC TE DISTRIBUTION

Despite the molecular data reported above, the link between the presence of TE vestiges in piRNA clusters and their silencing remained to be demonstrated. Bergman et al. (2006) proposed that β-heterochromatin TE nests could act as a trap for new TE invasions providing an “adaptive immunity” to the host genome. It could then be anticipated that different *Drosophila* lines have trapped certain TEs in piRNA clusters and not others, which would potentially explain their differential ability to repress distinct families of TEs. This was indeed what the primary genetic studies of *flam* had suggested for different *Drosophila* lines, displaying different capacities to repress or not the expression of *ZAM*, *Idefix,* and *gypsy*.

To test this possibility, Zanni et al. (2013) used the Rev line in which the mutation affecting *flam* releases the silencing exerted on *ZAM* and *Idefix*, but not on *gypsy*. The annotation of *flam* was refined in ISO1A, the line used to generate the genome sequence in which *ZAM*, *Idefix,* and *gypsy* are silenced. Several unknown properties of *flam* were highlighted in this study. We first found that among 52 different TEs present in the *flam* locus, the vast majority (49) are present as a unique copy. This observation supports a key prediction of the transposon trap model that postulates if a TE family is silenced as soon as it inserts *flam*, it should be present only once in the locus. This study also highlighted the high structural dynamics of this locus because numerous differences resulting from deletions, insertions or duplications were identified between different lines. In addition, sequence analysis of the *flam* TEs indicated that many of them correspond to TEs that recently inserted the locus. Among them, 12 new TEs were identified. Interestingly, eight of them were found closely related to TEs from *D. simulans*, *D. sechellia*, *D. yakuba,* or *D. erecta*, consistent with a recent origin from horizontal transfers that occurred between species belonging to the *melanogaster* subgroup (Bartolome et al., 2009).

To determine what underlies the difference between *Drosophila* lines that allow or restrict particular TEs to be mobilized, we compared the *flam* structure in ISO1A (restrictive for *ZAM*, *Idefix,* and *gypsy*) and Rev (restrictive for *gypsy* but not *ZAM* or *Idefix*). Importantly, a deletion of the region comprised between X:21638001 and 21684449 was found in Rev that encompasses the unique *ZAM* and *Idefix* copies present in *flam*. This observation provides the first evidence that a strict correlation exists between the presence or absence of TE sequences (i.e., *ZAM* and *Idefix*) within *flam* locus and repression or activity of that particular TE family. These new data highlight how structural variations in piRNA clusters impact the genomic TE distribution across the rest of the genome.

**FIGURE 5:**
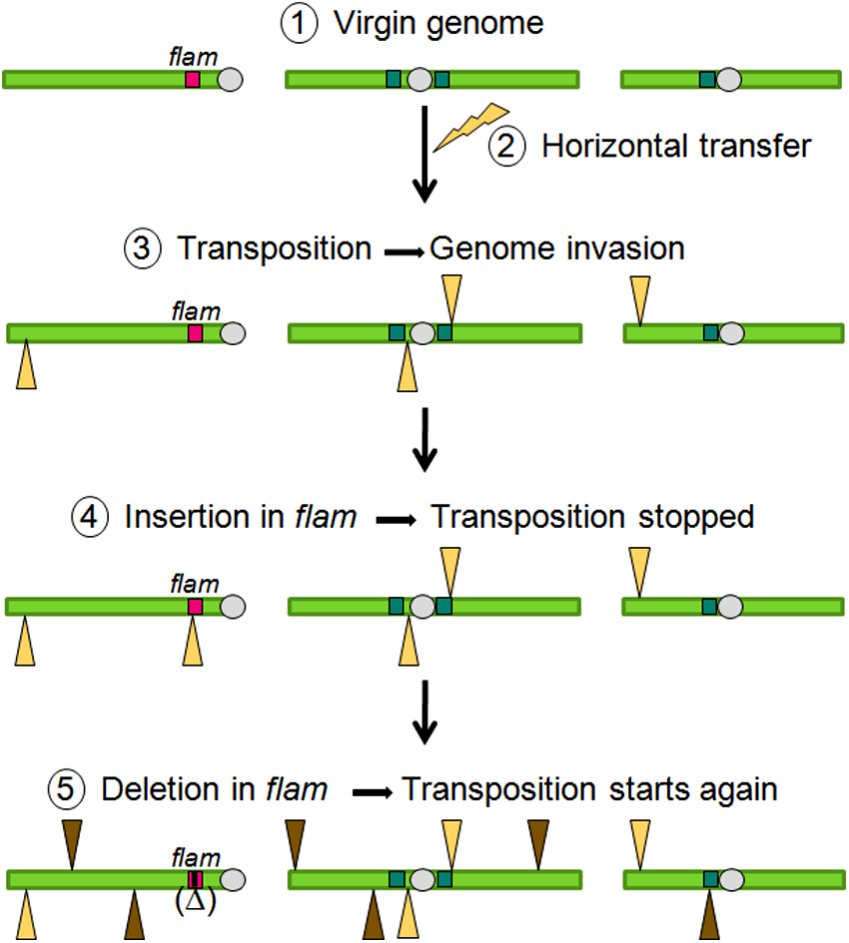
**Model of TE invasions, silencing, and remobilization**.

Overall, data obtained on *flam* fit with a model of TE invasion and its subsequent genomic control as follows (Figure 5). The best genetic background for a TE family to transpose is to enter a virgin genome in which no homologous sequence exists. In such a genome, no regulatory piRNAs are produced that are able to target the new TE. For that reason, horizontal transfer of a TE coming from another species increases the chances that a TE can invade a particular genome. After entering, the newly acquired TE starts replication cycles and its copies insert across the genome. Either by chance, because of relaxed selection, or because of active targeting, a new TE copy will eventually insert into a piRNA cluster. The pool of piRNA precursors produced by this locus will then be changed because of the presence of new sequences brought in by the new TE insertion. These new precursors, transferred to Dot COM and then processed in piRNAs in Yb-bodies will act in *trans* to silence their homologous copies. When this occurs, genomic stability is recovered. Due to their highly repetitive nature, piRNA clusters may subsequently undergo deletion events removing small or large portions of the locus. These deletions can remove TE sequences and may result in sudden bursts of transposition. Thus, periods of stability and instability in global TE dynamics will reflect the mutational events that affect piRNA clusters.

Conceptually, this dynamics of the *flam* locus provides an RNA-mediated adaptive immunity against TE invasions. Interestingly, this system in *Drosophila* shares striking resemblances with the CRISPR system developed by bacteria to fend off invaders (Barrangou and Marraffini, 2014). CRISPR loci (clustered regularly interspaced short palindromic repeats) are typically flanked by CRISPR-associated genes (Cas). The CRISPR-Cas system mediates immune defense involving sequence specific, RNA-mediated targeting of genetic invaders. The first step of the CRISPR-Cas protection occurs when new sequences derived from invading elements like viruses or plasmids are incorporated into the CRISPR locus. This locus is subsequently transcribed and processed into small interfering RNAs that guide Cas nucleases for specific cleavage of complementary sequences. This genome surveillance is thus triggered as soon as a TE, a virus or their derived sequences fall within the trap. It is interesting to note that, for both *flam* and CRISPR loci, these sequences remaining from invasions are transferred to the progeny in which they constitute genetic marks reflecting environmental changes over time.

After 40 years of data obtained on *flam*, it is interesting to measure how far we have gone since that time where heterochromatin was considered as a graveyard for TEs. Today, TEs and piRNA clusters in heterochromatin are thought to play fundamental roles in the organization and stability of genomes. The high structural dynamics of *flam* and potentially of the other piRNA clusters appears as a formidable evolutionary tool to remodel both euchromatic and heterochromatic regions, or even to play a role in speciation (Satyaki et al., 2014), by its ability to alternatively constrain or permit TE mobilization.

How far will further work on *flam* lead knowledge of heterochromatin function in the years to come?

## Conflict of Interest Statement

The authors declare that the research was conducted in the absence of any commercial or financial relationships that could be construed as a potential conflict of interest.
